# Lactonization of the Oncometabolite D-2-Hydroxyglutarate Produces a Novel Endogenous Metabolite

**DOI:** 10.3390/cancers13081756

**Published:** 2021-04-07

**Authors:** Raffaela S. Berger, Christian J. Wachsmuth, Magdalena C. Waldhier, Kathrin Renner-Sattler, Simone Thomas, Anuhar Chaturvedi, Hans-Helmut Niller, Elisabeth Bumes, Peter Hau, Martin Proescholdt, Wolfram Gronwald, Michael Heuser, Marina Kreutz, Peter J. Oefner, Katja Dettmer

**Affiliations:** 1 Institute of Functional Genomics, University of Regensburg, 93053 Regensburg, Germany; raffaela.berger@ukr.de (R.S.B.); chris_wachsmuth@freenet.de (C.J.W.); magdalena.waldhier@freenet.de (M.C.W.); Wolfram.Gronwald@ukr.de (W.G.); peter.oefner@ukr.de (P.J.O.); 2 Department of Internal Medicine III, University Hospital Regensburg, 93053 Regensburg, Germany; Kathrin.Renner-Sattler@klinik.uni-regensburg.de (K.R.-S.); Simone.Thomas@klinik.uni-regensburg.de (S.T.); Marina.Kreutz@klinik.uni-regensburg.de (M.K.); 3 Regensburg Center for Interventional Immunology, 93053 Regensburg, Germany; 4 Department of Hematology, Hemostasis, Oncology and Stem Cell Transplantation, Hannover Medical School, 30625 Hannover, Germany; Anuhar.Chaturvedi@biontech.de (A.C.); Heuser.Michael@mh-hannover.de (M.H.); 5 Institute of Medical Microbiology and Hygiene, University Regensburg, 93053 Regensburg, Germany; Hans-Helmut.Niller@klinik.uni-regensburg.de; 6 Department of Neurology and Wilhelm Sander-NeuroOncology Unit, Regensburg University Hospital, 93053 Regensburg, Germany; elisabeth.bumes@ukr.de (E.B.); peter.hau@ukr.de (P.H.); 7 Department of Neurosurgery, Regensburg University Hospital, 93053 Regensburg, Germany; Martin.Proescholdt@klinik.uni-regensburg.de

**Keywords:** D-2-hydroxyglutarate, IDH1/2 mutation, lactonization, acute myeloid leukemia

## Abstract

**Simple Summary:**

Somatic mutations in isocitrate dehydrogenase give rise to the excessive production and accumulation of D-2-hydroxyglutarate in certain malignancies. In addition to this well-described oncometabolite, we discovered a chemically related metabolite, namely 2-hydroxyglutarate-γ-lactone, which is derived directly from 2-hydroxyglutarate. This novel metabolite may impact the anti-tumor immune response.

**Abstract:**

In recent years, onco-metabolites like D-2-hydroxyglutarate, which is produced in isocitrate dehydrogenase-mutated tumors, have gained increasing interest. Here, we report a metabolite in human specimens that is closely related to 2-hydroxyglutarate: the intramolecular ester of 2-hydroxyglutarate, 2-hydroxyglutarate-γ-lactone. Using ^13^C_5_-L-glutamine tracer analysis, we showed that 2-hydroxyglutarate is the endogenous precursor of 2-hydroxyglutarate-lactone and that there is a high exchange between these two metabolites. Lactone formation does not depend on mutated isocitrate dehydrogenase, but its formation is most probably linked to transport processes across the cell membrane and favored at low environmental pH. Furthermore, human macrophages showed not only striking differences in uptake of 2-hydroxyglutarate and its lactone but also in the enantiospecific hydrolysis of the latter. Consequently, 2-hydroxyglutarate-lactone may play a critical role in the modulation of the tumor microenvironment.

## 1. Introduction

The deregulation of cellular energetics and metabolism is an important hallmark of cancer. The well-recognized “Warburg effect”, which describes the increased conversion of glucose to lactate in cancer cells (even in the presence of oxygen), was discovered many decades ago [1,2]. In recent years, several mutations that affect cellular metabolism were identified including the TCA cycle [3,4] and, in particular, the function of isocitrate dehydrogenase (IDH) [5,6]. IDH is a homodimeric enzyme with three different isoforms with distinct subcellular compartmentalization. IDH1, the cytosolic isoform, and IDH2, the mitochondrial isoform, are both NADP^+^-dependent, whereas mitochondrial IDH3 is NAD^+^-dependent and a core component of the TCA cycle. All isoforms catalyze the reversible oxidative decarboxylation of isocitrate to α-ketoglutarate [7]. For *IDH1* and *IDH2,* single base substitutions have been identified that result in a neo-enzymatic activity, i.e., the production of D-2-hydroxyglutarate (D-2-HG) from α-ketoglutarate [8]. Heterozygous *IDH1/2* mutations have been predominantly detected in gliomas, with an accumulation of D-2-HG in tumor tissue [9], and in acute myeloid leukemia (AML), with high serum levels of D-2-HG [10,11]. 2-HG production by mutated IDH1/2 is enantio-specific, with only the D-enantiomer being formed [8,10]. D-2-HG may contribute to tumorigenesis via different mechanisms. Due to its structural similarity to α-ketoglutarate, it exerts inhibitory effects on α-ketoglutarate-dependent enzymes [12,13,14]. The role of D-2-HG in prolyl hydroxylase (PHD) inhibition and, therefore, in HIF1α stabilization is still debated [12,14,15]. Furthermore, 2-HG is also taken up by cells in the microenvironment of *IDH1/2*-mutated tumor cells, e.g., immune [16,17,18] and stromal cells [19]. There, it triggers specific responses and finetunes the tumor environment. In addition, Chaturvedi et al. found that D-2-HG alone was not sufficient to induce transformation and leukemogenesis. It needed a collaborating oncogene (e.g., *HoxA9*) when administered to mice [20]. Similarly, mice transplanted with *HoxA9+IDH1mut*-expressing bone marrow cells had a significantly lower survival compared to *HoxA9* mice (wild-type (WT)-*IDH1/2*) injected with an oncogenic dose of D-2-HG. These data suggest that additional mechanisms to D-2-HG production play a role in the contribution of *mutIDH1* to leukemia development.

Recently, Strain et al. observed non-negligible amounts of *R*-2-HG-lactone (IUPAC: *R*-5-Oxo-2-tetrahydro-furancarboxylic acid), the intramolecular ester of *R*-2-HG (=D-2-HG), in urine specimens from *IDH1/2*-mutated brain tumor patients after the derivatization of 2-HG with (trimethylsilyl)diazomethane for enantioselective GC–MS analysis [21]. Here, we confirmed the endogenous occurrence of 2-HG-lactone in various biological specimens in the presence of *IDH1/2* mutations and investigated possible mechanisms for its formation. Furthermore, we describe important differences in cellular uptake between 2-HG and its lactone.

## 2. Materials and Methods

### 2.1. Materials and Standards

D- and L-2-hydroxyglutaric acid, *R*- and *S*-5-oxotetrahydro-2-furancarboxylic acid, pyridine, methyl chloroformate (MCF), and O-methylhydroxylamine hydrochloride were obtained from Sigma-Aldrich (Taufkirchen, Germany). *N*-methyl-*N*-(trimethylsilyl)-trifluoroacetamide (MSTFA) was purchased from Macherey-Nagel (Dueren, Germany). (2*R*)-2-hydroxyglutaric acid octyl ester sodium salt was bought from Toronto Research Chemicals (Toronto, ON, Canada), ^13^C_5_-L-glutamine was bought from CIL (Andover, MA, USA), and 2,3,3-D_3_-2HG was bought from C/D/N Isotopes Inc. (Pointe-Claire, QC, Canada). In all experiments, purified water from a PURELAB Plus system (ELGA LabWater, Celle, Germany) was used.

Deuterated 2-HG (510 µL of 1 mM 2,3,3-D_3_-2HG) was treated with HCl (120 µL of 4.5 N) at 100 °C for 30 min to form 2-HG-lactone-D_3_. This solution was evaporated to complete dryness and re-dissolved in water to yield a final concentration of 100 μM 2-HG-D_3_ and 2-HG-lactone-D_3_ (in sum) that was used as stable isotope-labeled standard.

### 2.2. Cell Culture Experiments and Sample Preparation

HT1080 (fibrosarcoma; ATCC-CCL-121) cells were cultured in DMEM supplemented with 10% fetal calf serum (FCS; Biochrom AG, Berlin, Germany), 1% penicillin–streptomycin (P/S; PAA; Pasching, Austria), and L-glutamine (PAN; 2 mM). HCT116 (colorectal carcinoma; HD 104-013, HD 104-019, and HD 104-020, Horizon Discovery, Waterbeach, UK) were cultured in RPMI (1640, PAN, Aidenbach, Germany) supplemented with 10 % FCS, 1% penicillin–streptomycin (P/S), and 2 mM L-glutamine. Mycoplasma contamination of cell lines was ruled out by regular tests.

For treatment experiments, cells were seeded into 6-well plates (Greiner Bio-One, Frickenhausen, Germany) at an equal cell density and kept under standard conditions overnight. Then, the supernatant was replaced with the respective medium containing, e.g., (2*R*)-2-hydroxyglutaric acid octyl ester sodium salt (Octyl-D-2-HG) at a concentration of 100 µM. Tracing experiments with ^13^C_5_-L-glutamine were performed with RPMI (+10% FCS and 1% P/S) without L-glutamine but supplemented with 2 mM ^13^C_5_-L-glutamine, and cells were incubated with the labeled substrate for 48 h.

For metabolite extraction, 50 µL of cell culture supernatant or serum were mixed first with 10 µL of a stable isotope-labeled internal standard solution and then with 200 µL of 100% methanol for protein precipitation.

For the determination of intracellular metabolite levels, adherent cells were washed twice with PBS, and then 10 µL of stable isotope-labeled standard (100 µM 2-HG/-lactone-D_3_) were added and the cells were scraped in 600 µL of 80% cold methanol. Culture wells were washed with another 400 µL of 80% methanol, and the wash was combined with the respective cell extract. Suspension cells were pelleted by centrifugation (150× *g* for 5 min) and thoroughly washed three times with 1 mL of PBS before the addition of internal standard and 600 µL 80% cold methanol.

All methanol-treated samples were stored at −80 °C overnight. For complete extraction, samples were centrifuged at 9560× *g* for 5 min at 4 °C, and supernatants were collected. The protein precipitates were washed twice with 100 µL of 80% methanol, and supernatants were combined in a glass vial. Samples were dried using an infrared vortex vacuum evaporator (CombiDancer, Hettich AG, Baech, Switzerland) and reconstituted in 50 µL of H_2_O. For tracing samples, the addition of the internal standard was omitted.

### 2.3. Human Primary Cells and Specimens

Peripheral blood mononuclear cells (PBMCs) were obtained from healthy donors by leukapheresis upon approval by the ethics committee of the University Hospital Regensburg (Ethic Vote July 2010 #09/066b and #09/066c). Monocytes were isolated from PBMCs by density gradient centrifugation over Ficoll/Hypaque followed by counterflow centrifugation elutriation. Monocytes were cultured on Teflon foils at a concentration of 1 × 10^6^ cells/1 mL in RPMI 1640 (Gibco, Waltham, MA, USA) for seven days to generate macrophages. The medium was supplemented with 2% pooled human serum, 2 mM L-glutamine (Biochrom, Berlin, Germany), 50 U/mL penicillin (Gibco, Waltham, MA, USA), and 50 U/mL of streptomycin (Gibco). For treatment experiments, macrophages were seeded at a concentration of 2 × 10^6^ cells per well in a 6-well plate in RPMI supplemented with 2% AB serum, 2 mM L-glutamine, and 50 U/mL penicillin/streptomycin and spiked with 1 mM D-2-HG or D-2-HG-lactone before being incubated for 24 h.

Serum specimens were collected at the Department of Internal Medicine III at the University Hospital Regensburg (Ethic Vote 05-097) and at the Hannover Medical School (Ethic Vote 936-2011). Serum was stored at −80 °C until a 50 µL-aliquot was extracted, as described above. AML blasts were isolated by standard Ficoll density separation from the peripheral blood or bone marrow of AML patients (Ethic Vote 05-097).

Frozen biopsies of human glioblastoma with known *IDH1/2* mutational status were provided by the Department of Neurosurgery at the University Hospital Regensburg (Ethic Vote 10-248-0219). The frozen tissues were immediately immersed into liquid nitrogen and stored at −80 °C until sample preparation. For tissue homogenization, between 30 and 150 mg of frozen tissue were transferred to Precellys 2-mL cups (pre-filled with 1.4 mm ceramic beads from Bertin Technologies, Montigny-le-Bretonneux, France), then 40 μL of an internal standard (20 μL for sample sizes < 50 mg) were added followed by 1000 μL of 80% ice-cold methanol and two cycles of homogenization using a Precellys homogenizer (Peqlab Biotechnologie GmbH, Erlangen, Germany). Further metabolite extraction followed the procedure described for cells, but different solvent volumes were used (tissue homogenates were washed twice with 500 μL of 80% methanol), and an additional wash with 600 μL of H_2_O was performed. The protein precipitate was not used for analysis. After the evaporation of the combined extract, samples were reconstituted in 200 μL of H_2_O (100 μL for sample sizes < 50 mg) prior to HPLC–MS/MS measurements. Metabolite concentrations in tissue extracts were normalized to tissue weight.

### 2.4. 2-HG and -Lactone Analysis by HPLC–MS/MS

HPLC–MS/MS analysis was performed as recently described [22,23] using an Agilent 1200 HPLC (Boeblingen, Germany) coupled to an API 4000 QTRAP (AB SCIEX, Darmstadt, Germany). A Discovery HS F5-3 HPLC column (15 cm × 2.1 mm, 3 µm; Supelco, Bellefonte, PA, USA) was used. Gradient elution was performed with mobile phase A consisting of 0.1% formic acid (FA) in water (*v*/*v*) and 100% acetonitrile (ACN) as mobile phase B. The gradient for chromatographic separation started with 0% B (200 μL/min), increased to 100% B from 6.50 min to 8 min at 350 μL/min, and stayed at 100% B for 2 min, followed by a reconditioning of the column at the starting condition for 8 min. Detection was carried out in negative ionization MRM (multiple reaction monitoring) mode using the following ion transitions: *m*/*z* 147.1 [M − H]^−^ to *m*/*z* 84.8 for 2-HG, *m*/*z* 150.1 [M − H]^-^ to *m*/*z* 87.8 for 2-HG-D_3_, *m*/*z* 128.8 [M − H]^−^ to *m*/*z* 100.8 for 2-HG-lactone, and *m*/*z* 131.8 [M-H]^−^ to *m*/*z* 87.9 for 2-HG-lactone-D_3_. Metabolite quantification was achieved using a calibration curve with the areas of target compounds normalized by the areas of the corresponding stable isotope-labeled standards. Metabolite concentrations were normalized to total protein amount, or tissue weight.

For isotope tracing, transitions for all isotopologues were set up (e.g., 2-HG (M + 0) 147.1 *m*/*z* → 84.8 *m*/*z*, 2-HG (M + 1.1) 148.1 *m*/*z* → 85.8 *m*/*z*, and 2-HG (M + 1.0) 148.1 *m*/*z* → 84.8 *m*/*z*). Peak areas of all isotopologues were corrected for the natural abundance of stable isotopes and impurities of the tracer using the R-package IsoCorrectoR [24].

### 2.5. Chiral GC–MS Analysis

The dried serum extract (see above) was reconstituted in 100 µL of water and subjected to derivatization with MCF/methanol as previously described [25]. Derivatives were extracted with chloroform, and a 1 µL aliquot was injected into the GC-TOFMS. A 450-GC (Bruker Daltonics GmbH, Bremen, Germany) equipped with a PAL COMBI-xt autosampler (CTC Analytics, Zwingen, Switzerland) was coupled to a microTOF orthogonal acceleration TOF mass spectrometer (Bruker Daltonics) via an APCI II source [26,27,28] using the following conditions: capillary voltage: −3000 V; end plate offset: −500 V; corona discharge needle,:+4000 nA; sheath gas: nitrogen at 2.5 bar; dry gas: nitrogen; temperature: 150 °C; flow: 2.0 mL/min. Separation was performed on an Rt-γDEXsa (30 m × 0.25 mm ID × 0.25 µm film thickness) column using helium as carrier gas with a flow rate of 2 mL/min. A sample volume of 1 µL was injected splitless at an injection temperature of 250 °C. The temperature program started at 70 °C, followed by a hold for 1 min; then, temperature was ramped at 5 K/min to 190 °C and finally held for 7 min.

### 2.6. Measurement of Protein

The protein content of cell pellets was determined using the FluoroProfile^®^ Protein Quantification Kit (Sigma Aldrich), which included a bovine serum albumin standard for preparing a standard curve. Later, the dye was replaced with the fluorescent dye SERVA Purple (Serva, Heidelberg, Germany), and the assay buffer was prepared from water, dye, and a buffer (25% acetonitrile, 3% SDS, and 200 mM sodium bicarbonate in water) (8:1:1). Cell pellets were lysed in a 20 mM NaH_2_PO_4_ buffer containing 1.2% SDS. The assay was performed in black 96-well plates (Greiner Bio-One). Samples were diluted with water, mixed with the assay buffer, and incubated for 1.5 h. Fluorometric analysis was performed at an excitation wavelength of 485 nm and an emission wavelength of 600 nm.

### 2.7. NMR

#### 2.7.1. Sample Preparation for NMR Analysis

As the presence of macromolecules gives rise to broad background signals that hinder the analysis of metabolite signals, serum specimens were filtered prior to analysis. To this end, serum samples were thawed, thoroughly shaken, and ultra-filtered employing Nanosep centrifugal devices (Pall Corporation, Port Washington, NY, USA) with a three kDa molecular weight cutoff.

Then, 400 μL of the filtrate or aqueous standard were transferred into a 5 mm NMR tube (Bruker BioSpin GmbH, Rheinstetten, Germany), followed by the addition of 200 μL of a potassium phosphate buffer at pH 7.4 and 50 μL of 0.75% (w) 3-trimethylsilyl-2,2,3,3-tetradeuteropropionate (TSP) dissolved in deuterium oxide as an internal standard.

#### 2.7.2. NMR Measurements

Following established protocols [29], 1D ^1^H and 2D ^1^H-^13^C TOCSY spectra were acquired on a 600 MHz Bruker Avance III (Bruker) employing a triple resonance (^1^H, ^13^C, ^15^N, and ^2^H lock) cryogenic probe equipped with *z*-gradients and an automatic, cooled sample changer. By employing TopSpin 3.1 (Bruker BioSpin GmbH, Rheinstetten, Germany), spectra were semi-automatically Fourier-transformed and phase-corrected, followed by polynomial baseline correction.

#### 2.7.3. Analysis of NMR Data

Amix viewer V3.9.13 (Bruker BioSpin) was used for the manual inspection of spectra. Signals in the spectra of pure reference compounds were assigned by employing predicted ^1^H chemical shifts and signal intensities obtained by ChemDraw (PerkinElmer, Waltham, MA, USA). In serum spectra, 2-HG and its lactone were identified by overlay with the previously assigned reference spectra. The phosphate buffer used in both reference and serum spectra ensured that no pH-dependent signal shifts occurred. Furthermore, as filtered serum was used, no shifts in the position of the TSP reference signal due to protein binding occurred.

### 2.8. Data Analysis and Statistics

HPLC–MS/MS data were analyzed using Analyst (version 1.6.2, AB Sciex), GC–MS data using MassHunter Workstation Software (version B.07.01, Agilent) and MassLynx 4.1 (Waters Inc., Milford, MA, USA). Further data analysis was performed using MS Excel (two-tailed *t*-test) and R (version 1.2.5003, ANOVA and post-hoc tests). Molecular structures were drawn using ChemDraw (PerkinElmer).

## 3. Results and Discussion

### 3.1. 2-HG-Lactone Is a Novel Endogenous Metabolite

**Figure 1:**
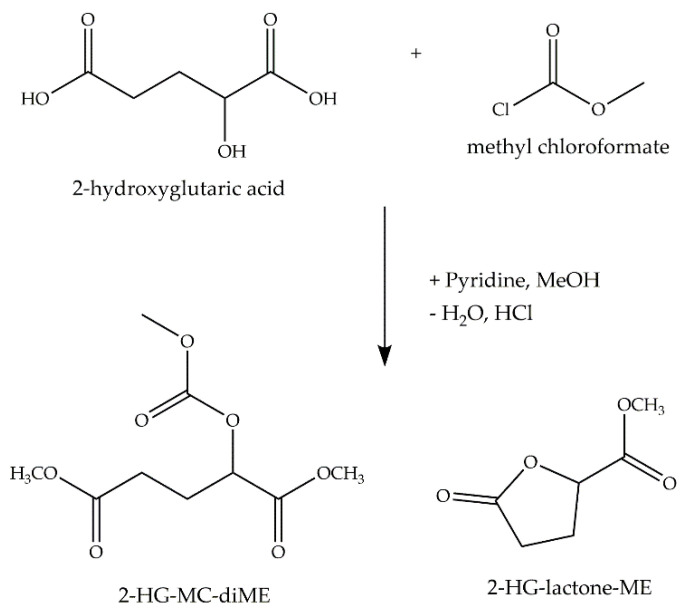
Reaction scheme for methyl chloroformate (MCF) derivatization of 2-HG. Derivatization of 2-HG with MCF/methanol yields two main derivatives, the three-fold derivative of the open 2-HG and the one-fold derivative of the 2-HG-lactone (MC: methoxycarbonyl group; ME: methyl ester group).

We implemented a GC–MS method for the chiral analysis of 2-HG after derivatization with MCF/methanol (Figure 1). In addition to the expected signals for the D and L derivative of 2-HG (2-((O-methoxycarbonyl)oxy)glutaric acid dimethyl ester), we detected two signals that were identified as methyl esters of the D/L-2-hydroxyglutaric acid lactone.

**Figure 2:**
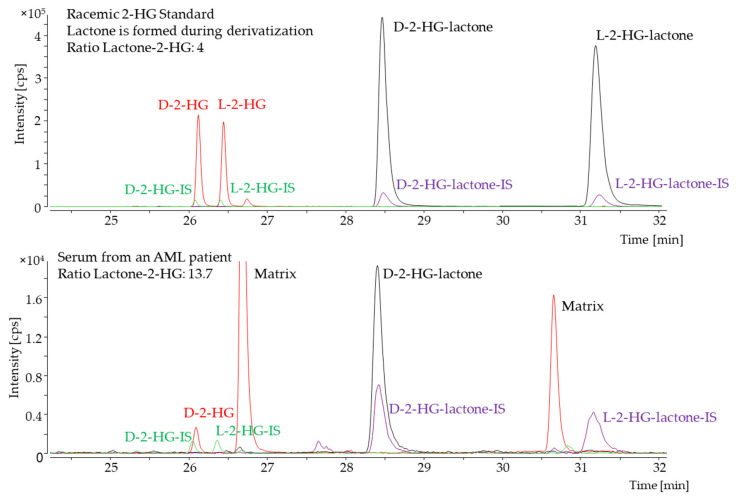
Chiral GC–MS analysis. The red trace shows the extracted ion chromatogram for the D- and L-enantiomer of the 2-HG derivative. The corresponding stable isotope-labeled internal standard (IS) is shown in green. The extracted ion chromatogram for the D- and L-enantiomer of the 2-HG-lactone derivative is shown in black, and the corresponding IS-trace in purple. The upper panel shows a racemic D/L-2-HG standard, and the lower panel shows a serum sample of an acute myeloid leukemia (AML) patient with an *IDH2-R140Q* mutation. For standard solutions, the ratio of each enantiomer of 2-HG and -lactone was found to be consistent. For the serum sample, both L-enantiomers were hardly detectable, while the ratio of D-lactone/2-HG was found to be increased for the unlabeled analyte.

**Figure 3:**
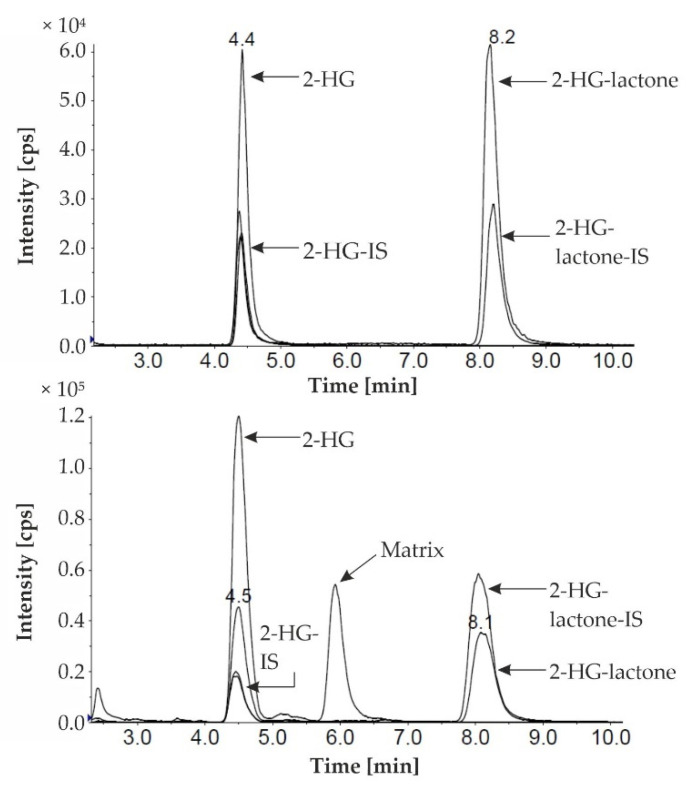
HPLC–MS/MS analysis of a mixture of 2-HG and 2-HG-lactone standard solutions (upper chromatogram) and a serum sample (lower chromatogram) from an AML patient with an *IDH(1-R132L)* mutation. 2-HG/-lactone marks the traces of the endogenous metabolites, and -IS marks the traces of the stable isotope labeled internal standards (2-HG/-lactone-D_3_).

Lactone formation during the derivatization of 2-HG has already been described by others as a side-product of the derivatization reaction or due to the acidification of the sample [21,30,31]. We observed a stable ratio of 4:1 (lactone/2-HG) of the two derivatives when standard solutions were analyzed. Contrarily, in some serum samples of patients with a *IDH1/2* mutation, the intensity of the D-2-HG-lactone derivative was increased in comparison to the D-2-HG derivative (Figure 2), while both L-enantiomers were hardly observable. Hence, we hypothesized that the D-2-HG-lactone is also an endogenous metabolite. To analyze this metabolite further, we used an HPLC–MS/MS method that was previously implemented for the achiral analysis of 2-HG. Gentle sample preparation using methanol precipitation and no derivatization should have prevented lactonization. Accordingly, we were able to detect two peaks for 2-HG and 2-HG-lactone. The difference in mass between the two compounds is equal to a water molecule (18 Dalton), as water loss is a common phenomenon during electrospray ionization. Therefore, the unambiguous differentiation of the two compounds based solely on their mass was not feasible. Since the two metabolites were chromatographically separated, we could corroborate 2-HG-lactone as an endogenous metabolite (Figure 3).

**Figure 4:**
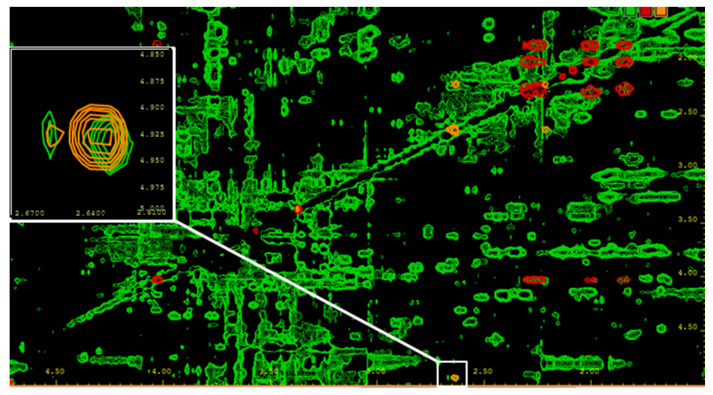
2D TOCSY of a serum sample from an AML patient with an *IDH2-R140Q* mutation. For the removal of macromolecules, the serum was filtered using a Nanosep filtering device with a 3 kDa cutoff. Signal assignment was based on comparison with reference spectra of pure compounds. Green = serum sample; red = 2-HG reference spectrum; and orange = 2-HG-lactone reference spectrum. Besides overlapping signals for both compounds, unique signals are present, as shown in the white box for the 2-HG-lactone, and allow for the discrimination of the two metabolites. In NMR spectra of probands carrying no *IDH1/2* mutation, neither 2-HG nor its lactone could be detected.

NMR analysis confirmed the finding of D-2-HG-lactone as an endogenous metabolite in serum samples from AML patients with elevated D-2-HG levels as a consequence of an *IDH1/2* mutation (Figure 4).

**Figure 5:**
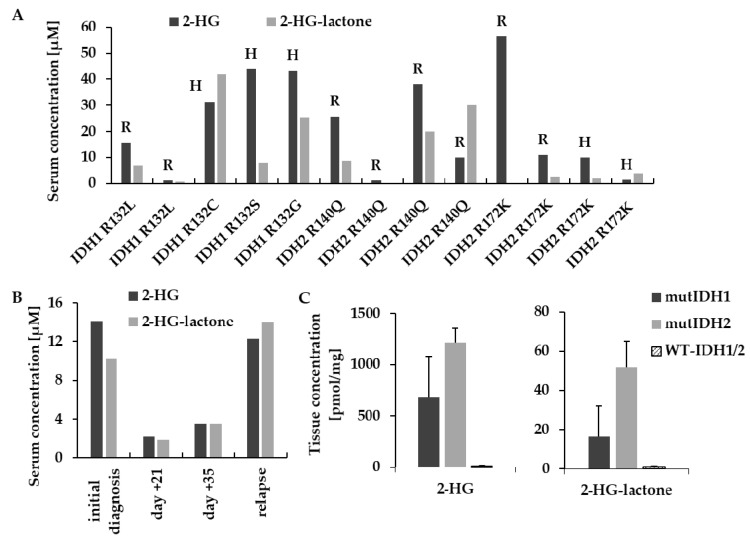
Analysis of 2-HG and 2-HG-lactone in biological specimens. (**A**) Serum samples from AML patients with different *IDH1/2* mutations collected at two different sites (R = Regensburg; H = Hannover) that were drawn at different time points after diagnosis. (**B**) Exemplary time course of serum levels of 2-HG and 2-HG-lactone in an *IDH2-R140Q*-mutated AML patient. Initial response and subsequent treatment failure caused 2-HG and 2-HG-lactone serum levels to drop and rise in unison. (**C**) 2-HG and 2-HG-lactone levels in glioblastoma tissue samples (mut*IDH1*: n = 6; mut*IDH2*: n = 3; WT-*IDH1/2*: n = 5; 2-HG: ANOVA *p* = 2.86 × 10^−4^; post-hoc test = Dunnett WT vs. mut*IDH1 p* = 2.12 × 10^−3^; WT vs. mut*IDH2 p* = 9.64 × 10^−5^; 2-HG-lactone ANOVA *p* = 1.25 × 10^−3^; WT vs. mut*IDH1 p* = 9.19 × 10^−2^; Wtvs. mut*IDH2 p* = 3.9 × 10^−4^).

The two metabolites were further quantified by HPLC–MS/MS in AML serum samples collected at two different clinical sites. 2-HG and 2-HG-lactone were present at varying ratios (up to three-fold 2-HG-lactone; Figure 5A), but 2-HG-lactone was never found alone. Further, the serum levels of 2-HG-lactone reflected tumor load, as already reported for D-2-HG [32,33,34], over the course of treatment (Figure 5B).

In addition to AML serum, we also analyzed glioblastoma tissue samples with and without *IDH1/2* mutation. The lactone was detected in the samples of patients with *IDH1/2* mutation, but the average level of 2-HG-lactone relative to 2-HG was much lower with ~2% of sum of 2-HG and 2-HG-lactone; Figure 5C. The same was true for AML blasts isolated from either bone marrow or peripheral blood from an *IDH2-R140Q*-mutated AML patient (n = 1, preliminary results; data not shown). Though 2-HG-lactone was detected in the serum of the patient, levels in the blasts were low (0.1–0.2% of sum of 2-HG and 2-HG-lactone). Table S1 summarizes the findings in human specimen.

We hypothesized that 2-HG-lactone is directly formed from 2-HG by either enzymatic or spontaneous intramolecular esterification. To date, in humans, only enzymes that hydrolyze lactones, such as 6-phosphogluconolactonase (which catalyzes the second step of the pentose phosphate pathway [35]), are known. However, for other organisms, enzymes catalyzing a lactonization reaction have been described. One prominent example is the gluconolactonase (*SMP30*, regucalcin) involved in the synthesis of vitamin C in mice [36]. For *Burkholderia sp. R-711*, a lactonase that acts on D-2-HG-lactone, has been described, but no lactone formation has been detected [37].

To test whether the lactone is formed by mutant *IDH1/2*, we incubated recombinant *IDH1-R132H*, *IDH2-R172K*, and WT-*IDH1* enzymes in vitro (see Table S2 for experimental details) with α-ketoglutarate, and assay aliquots were analyzed by HPLC–MS/MS. While 2-HG was produced, the lactone was not identified (Figure S1). We further investigated whether hepatic metabolism was involved in lactone formation by incubating microsomes (human microsomes and S9-fraction; Life Technologies) and primary hepatocytes with 2-HG, but no 2-HG-lactone production was observed (experimental details see Table S2). Furthermore, the incubation of 2-HG in serum from healthy donors and an *IDH*-mutated AML patient did not result in 2-HG-lactone production (data not shown).

We also tested cell lines carrying an *IDH1/2* mutation for lactone production. HT1080, a fibrosarcoma cell line carrying an *IDH1-R132C* mutation, showed elevated 2-HG levels but no 2-HG-lactone production. Additionally, we used an HCT116 cell panel consisting of the parental line with WT-*IDH1/2* and three cell lines with different *IDH1/2* mutations (*IDH1-R132H, IDH2-R172K,* and *IDH2-R140Q*). Interestingly, the mut*IDH1/2* HCT116 cell lines produced not only 2-HG but also 2-HG-lactone (Figure S2).

### 3.2. 2-HG-Lactone Is Formed from 2-HG

**Figure 6:**
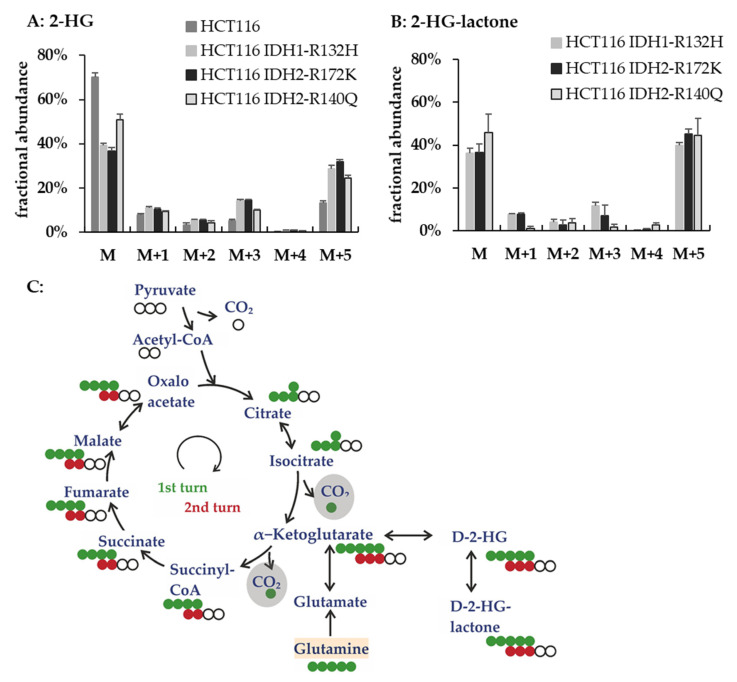
Tracing experiments in *IDH1/2*-mutant HCT116 cell lines revealed a high exchange between 2-HG and 2-HG-lactone. Fractional abundance of isotopologues of 2-HG (**A**) and 2-HG-lactone (**B**) for *IDH1/2*-mutant HCT116 cells after cultivation with U-^13^C-glutamine for 48 h (M is the unlabeled isotopologue, and M + X indicate the isotopologues with increasing numbers of ^13^C). For the parental cell line, no results for 2-HG-lactone are shown because this metabolite was not present in those samples. (**C**) Scheme of TCA-derived metabolites in U-^13^C-glutamine tracer analysis: full circles depict ^13^C atoms (green derived directly from U-^13^C-glutamine and red after one turn in the TCA cycle), and open circles indicate ^12^C atoms derived from unlabeled precursors, e.g., pyruvate which is the end product of glycolysis. U-^13^C-glutamine was added to the cell culture medium and refueled the TCA by anaplerotic reactions to form α-ketoglutarate. Thus, after each turn in the TCA cycle, two labeled ^13^C were lost in form of CO_2_ and were substituted by the unlabeled C_2_-body of acetyl-CoA. The expected labeling pattern after one or two cycles through the TCA are shown in green and red, respectively. Hence, one can mainly expect the M + 5/M + 3 of 2-HG in HCT116 cell extracts cultured with 2 mM U-^13^C-glutamine.

In the following sections, we describe different cell culture experiments that were performed to investigate the 2-HG lactonization (summarized in Table S2). To elucidate whether 2-HG was the endogenous precursor of 2-HG-lactone, we incubated the HCT116 cell line panel with U-^13^C-glutamine for 48 h. The ^13^C-labeled glutamine was converted to 2-HG via glutamate and α-ketoglutarate (αKG). Figure 6 shows the labeling pattern of 2-HG and 2-HG-lactone obtained from the tracing experiments and a scheme of the expected label distribution when glutamine is metabolized via the TCA cycle.

The isotopologue distributions obtained for 2-HG and 2-HG-lactone were very similar. This indicated a high exchange between the two metabolites (Figure 6A,B). The most abundant isotopologue next to the unlabeled (M + 0) was the (M + 5) isotopologue, which is directly formed from ^13^C_5_-glutamine without αKG being fed into the TCA cycle. In conclusion, these experiments provided evidence that 2-HG-lactone is indeed directly formed from 2-HG by intramolecular ester formation.

### 3.3. Mechanisms for Lactone Formation

**Figure 7:**
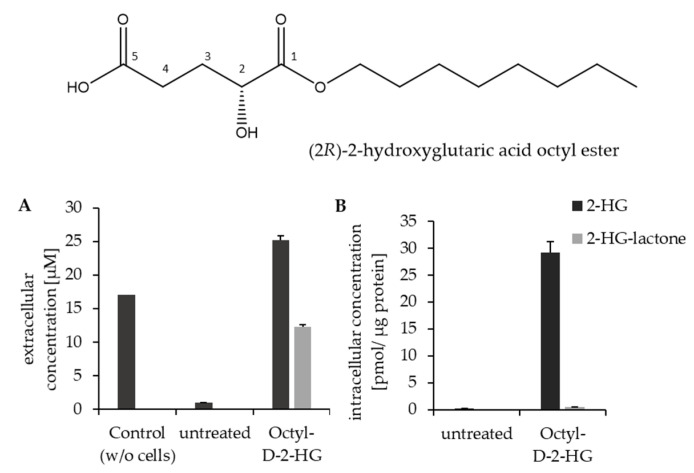
Parental HCT116 cells were treated with 100 µM octyl-D-2-HG and compared to untreated cells. (**A**) 2-HG and 2-HG-lactone levels in cell culture supernatant and (**B**) intracellular levels (n = 3). This experiment showed that the hydrolysis of octyl-D-2-HG already takes place in a medium releasing free 2-HG, but lactone formation is only found in the presence of cells. 2-HG, but no 2-HG-lactone, was detected in blank medium incubated with octyl-D-2-HG but without cells (left bar). The percentage of 2-HG-lactone from the sum of 2-HG and lactone was higher in the medium than in the cell extracts. Intracellular 2-HG-lactone concentration was close to LLOQ.

To further investigate whether lactone formation is dependent on the mutational status of *IDH1/2*, parental HCT116 cells were cultured in the presence of (2*R*)-2-hydroxyglutaric acid octyl ester (octyl-D-2-HG). The octyl ester is a cell-membrane-permeable derivative of 2-HG that results in high intracellular 2-HG levels. Treating parental HCT116 (WT-*IDH1/2*) cells with 100 µM octyl-D-2-HG resulted in intracellular 2-HG amounts that were comparable to the endogenous levels observed in *IDH1-R132H*- and *IDH2-R172K*-mutated HCT1216 cell clones (sees Figure 7A and Figure S2B). Furthermore, we were able to detect 2-HG-lactone in the cell extracts, but, interestingly, most of the lactone was found in the cell culture supernatant (Figure 7). Therefore, we conclude that HCT116 cells can form 2-HG-lactone out of 2-HG as long as sufficient intracellular 2-HG amounts are available. In untreated parental HCT116, only low 2-HG amounts were present, which may not have sufficed to cause 2-HG-lactone formation, or the lactone amounts were too low to be detected above the lower limit of quantification (LLOQ) with the method used for analysis. Note that octyl-D-2-HG itself was not quantified, as measurements were performed in MRM mode, detecting only free 2-HG and 2-HG-lactone.

**Figure 8:**
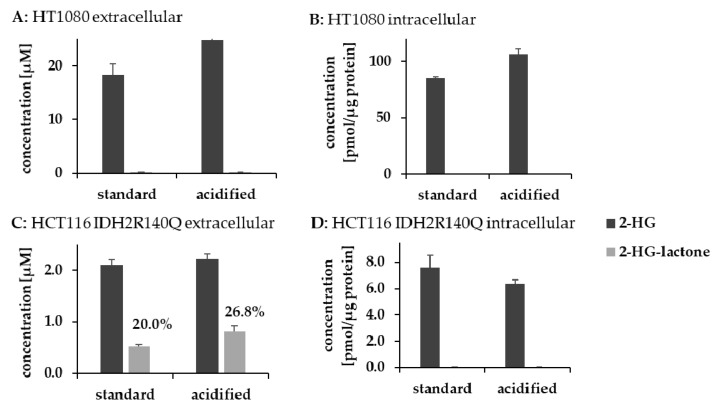
Impact of pH on 2-HG-lactonization in cells. *IDH1/2* mutant cells were cultured under acidified conditions to investigate the impact of pH on 2-HG lactonization. (**A**,**B**) Extra- and intracellular concentrations of 2-HG and 2-HG-lactone after 6 h for HT1080 cells; (**C**,**D**) extra- and intracellular concentrations of 2-HG and 2-HG-lactone after 6 h for HCT116 *IDH2-R140Q*. 2-HG-lactone was below the LLOQ in cell extracts. (n = 2–3; error bars indicate SD; and percentages represent amounts of 2-HG-lactone relative to the sum of 2-HG and 2-HG-lactone).

Octyl-D-2-HG treatment was also performed with nine additional WT-*IDH1/2* cell lines of different origins (Figure S3). Interestingly, upon octyl-D-2-HG treatment, all cell lines were capable of 2-HG lactonization, with most of the lactone found extracellularly. However, the detected lactone concentrations (whether intra- or extra-cellularly) varied across cell lines. This might have been caused by varying activities of the cellular esterases responsible for the intracellular hydrolysis of 2-HG-lactone. The observed 2-HG-lactone was probably at least partially formed in the process of octyl-D-2-HG hydrolysis, which might explain why lactone formation was observed in HT1080 cells after octyl-D-2-HG but not without treatment, although high 2-HG levels were endogenously present (Figure 8A,B). We suppose that this could be explained by the fact that during the intracellular ester hydrolysis of the octyl-D-2-HG by intracellular esterases, a negatively charged transition state with the negative charge located at the double-bonded oxygen of the (C1) carboxyl-group is obtained [38]. This negative charge also leads to an increased negative polarization of the neighboring OH group at position (C2), which in turn will favor a nucleophilic attack of this OH group on the opposed carboxylic carbon (C5) and thereby promote the lactonization of 2-HG.

### 3.4. Lactonization Is Favored under Acidic Conditions

Since acidic conditions are known to catalyze esterification, it is not surprising that the formation of 2-HG-lactone is facilitated at a low pH. Cancer cells are known to produce large amounts of lactic acid, which also causes the pH to drop. However, pH is tightly regulated in vivo. The intracellular pH of cancer cells is maintained within a range between 7.1 and 7.2 [39], and even in cancer patients, blood pH is not significantly different from that of healthy human subjects [40]. Nevertheless, we tested the pH dependency of 2-HG-lactone formation. We incubated 2-HG overnight in an HEPES buffer (20 mM) at 37 °C under different pH conditions and quantified 2-HG-lactone formation. Already at a pH of 7, spontaneous 2-HG-lactone formation could be detected (Figure S4). Lactone formation increased exponentially with a decreasing pH. At a pH of about 5.5, two percent of the 2-HG underwent intramolecular esterification, which was still a fairly low percentage. As the pH in serum or a cell culture medium is controlled by buffer systems, lactone formation should not take place in significant amounts there. AML cells are characterized by a high glycolytic activity [41,42,43], and the pH in the bone marrow of those patients is reduced. For leukemic rats, the bone marrow pH was reported to drop from 6.9 to 6.5 [44]. However, measuring bone marrow pH is not trivial. Assuming that the pH in the environment of AML cells is also lower, it might be possible that 2-HG lactonization takes place in the bone marrow before 2-HG and its lactone are flushed into the peripheral blood. However, the question of how 2-HG lactonization is explained in the *IDH1/2*-mutated HCT116 cell lines remains. We therefore tried to evaluate the impact of environmental pH on 2-HG lactonization in *IDH1/2*-mutated cell lines. HCT116 *IDH2-R140Q* and HT1080 *IDH1-R132C* cells were cultured at different pH values. To mimic lower pH conditions, we acidified the medium to ~pH 6.5 by adding HCl and then cultured the cells for 6 h. Within this period, the cells were still vital. With HCT116 *IDH2-R140Q* cells, which already produce 2-HG-lactone under normal conditions, we indeed observed an increase in extracellular 2-HG-lactone concentrations (which might have been a pH stabilizing effect; two-tailed *t*-test: *p* = 0.011; Figure 8). However, for HT1080 cells, lactonization could not be initiated by lowering the extracellular pH. Consequently, we assume that pH is not the only factor regulating 2-HG lactonization.

### 3.5. Highlighting the Difference between 2-HG and 2-HG-Lactone

**Figure 9:**
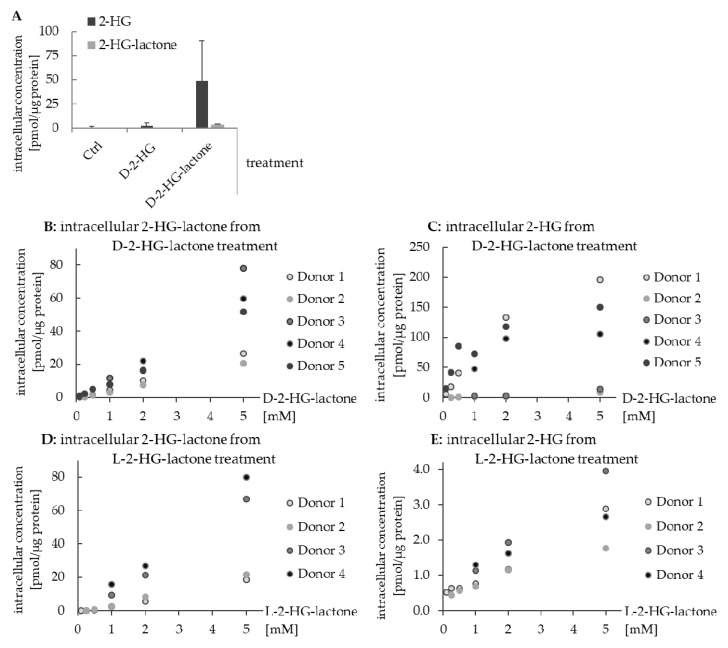
Difference in the uptake and hydrolysis of 2-HG and 2-HG-lactone by human macrophages. (**A**) Intracellular levels of 2-HG and 2-HG-lactone in macrophages of three donors after treatment with either 1 mM D-2-HG or 1 mM D-2-HG-lactone in comparison to untreated cells (error bars represent SD and are rather large because of donor variability). (**B**,**D**) intracellular 2-HG-lactone concentration after the treatment of macrophages with increasing concentrations of D-2-HG-lactone and L-2-HG-lactone, respectively. (**C**,**E**) Intracellular 2-HG concentration after the treatment of macrophages with increasing concentrations of D-2-HG-lactone and L-2-HG-lactone, respectively. In donors 2 and 3, the hydrolysis of D-2-HG-lactone was obviously less efficient than in the other donors, but was still on a higher level than for L-2-HG-lactone.

Though 2-HG and 2-HG-lactone can be converted into each other, it is still important to consider them as two separate metabolites. This becomes evident in human macrophages treated with 1 mM D-2-HG or D-2-HG-lactone. As shown in Figure 9A, the uptake of D-2-HG from the culture medium was low, while D-2-HG-lactone was readily taken up and hydrolyzed inside the cell. Hence, both the lactone and 2-HG were detected in the cell extract. The sum of 2-HG-lactone and hydrolyzed 2-HG obtained after treatment with 1 mM D-2-HG-lactone exceeded the 2-HG level obtained by treatment with D-2-HG by a factor of 20 (Figure 9A).

The difference in the cellular uptake of 2-HG and 2-HG-lactone is most likely the result of their transport by different membrane transporters: 2-HG is transported via OAT1/4 [45] and dicarboxylate transporter 3 (*SLC13A3*) [46], while 2-HG-lactone is assumed to be transported by MCTs (monocarboxylate transporters). The difference in the uptake of D-2-HG and D-2-HG-lactone could also be observed for other cell lines (see Figure S5). We therefore concluded that intracellular concentrations of D-2-HG-lactone and D-2-HG, e.g., in immune cells in the tumor microenvironment, can drastically vary depending on the concentration of D-2-HG-lactone (Figure 9B,C). D-2-HG is known to affect immune cell metabolism and functional properties such as interleukin secretion [16,17,18,47,48]. Hence, D-2-HG-lactone is also expected to impact the immune response and oncogenesis, which needs to be further investigated. This highlights the importance of considering 2-HG-lactone as an additional factor in processes affected by 2-HG.

In the course of the macrophage experiments, we also found an enantiospecific difference in lactone hydrolysis. We incubated the cells not only with D-2-HG-lactone but also with L-2-HG-lactone. The uptake of D-/L-2-HG-lactone resulted in comparable intracellular levels of the respective enantiomer (Figure 9B,D). However, intracellular 2-HG levels resulting from lactone hydrolysis were considerably higher after treatment with D-2-HG-lactone (Figure 9C,E). This suggests enantiospecific lactone hydrolysis by enzymes that need to be elucidated in future studies.

Concerning the effects evoked by 2-HG-lactone, it is important to elucidate the mechanism of 2-HG-lactone formation. The administration of 2-HG-lactone can be used to investigate its effects. However, caution is advised because the intracellular hydrolysis of 2-HG-lactone hampers the conduction of experiments. To this end, results from 2-HG-lactone treated cells represent a mixture of 2-HG and 2-HG-lactone effects.

## 4. Conclusions

In this report, we studied the varying ability of cells to catalyze the lactonization of D-2-HG. However, the detailed mechanism of 2-HG-lactonization remains unclear. In general, 2-HG-lactone accumulated extracellularly, e.g., in the cell culture supernatant or sera from AML patients. In contrast, intracellular levels in cell lines, AML blasts, and tumor tissues were low. We showed that even at a neutral pH, small amounts of 2-HG-lactone were spontaneously formed from 2-HG under in vitro conditions, with the equilibrium being on the 2-HG side. Monocarboxylate transporters are likely to transport the formed lactone across the cell membrane to the extracellular space, where it will accumulate, while 2-HG remains in the cell due to a lower export rate. Through this, the intracellular equilibrium is perturbed, leading to the continued formation of 2-HG-lactone. Consequently, transport processes across the cell membrane indirectly lead to 2-HG lactonization.

Further efforts are necessary to unravel the origin and the biological effects of 2-HG-lactone, which is likely to increase our understanding of tumorigenesis in *IDH1/2*-mutant cancers.

## Data Availability

The data presented in this study are available on request.

## Supplementary Materials

The following are available online at https://www.mdpi.com/article/10.3390/cancers13081756/s1. Supplemental Tables, Table S1. Overview of human specimens used to quantify 2-HG and its lactone, Table S2: Overview of cell lines used in this study, Supplemental Results, Table S3: Experimental details about in vitro lactone formation, Supplemental Figures, Figure S1: Enzyme assay using recombinant WT-*IDH1* and mut*IDH1/2* and α-ketoglutarate as substrate. Figure S2: Extracellular and intracellular 2-HG and 2-HG-lactone concentrations in the HCT116. Figure S3: 2-HG and 2-HG-lactone levels in the supernatants and cell extracts of octly-2-HG treated cells. Figure S4: Spontaneous, non-enzymatic 2-HG lactonization under acidified conditions. Figure S5: Intracellular concentrations of 2-HG and its lactone in C7H2 and MCF7 cells after treatment with D-2-HG or D-2-HG-lactone.
