# Supplementary material for: Lactonization of the Oncometabolite D-2-Hydroxyglutarate Produces a Novel Endogenous Metabolite
Source: Cancers (Basel). 2021 Apr 7;13(8):1756. doi: 10.3390/cancers13081756 (PMC8067704; doi:10.3390/cancers13081756)
Supplement: Supplementary file 1 [file cancers-13-01756-s001.pdf]

cancers

MDPI

Supplemental Tables

Table 1. Overview of human specimens used to quantify 2-HG and its lactone.

Sample Type	Mutational status	#	Endogenous 2-HG-lactone
**Glioma tissue**	wt	5	no
	mutIDH1	6	2 % of sum of 2-HG and 2-HG-lactone
	mutIDH2	3	
**AML serum**	mutIDH1	5	Quite different amounts
	mutIDH2	8	Quite different amounts
	IDH2-R140Q	Series of 4 time points	Reflecting tumor load
**Blasts**	*IDH2-R140Q*	1 (Bone marrow derived)	0.1-0.2 % of sum of 2-HG and 2-HG-lactone
		1 (peripheral)	no
**Macrophages**	wt	6	no

Table 2. Overview of cell lines used in this study.

Cell line/ panel (origin)	Mutational status	Endogenous production		**Experimental details & results**	
**HCT116 (colorectal carcinoma)**	wt	baseline	no	$^{13}C_5$-glutamine-tracing analysis	
	IDH1-R132H	yes	yes		
	IDH2-R172K	yes	yes		
	IDH2-R140Q	yes	yes		Cultured under acidic conditions → higher amounts of 2-HG-lactone were detected
**HT1080 (fibrosarcoma)**	*IDH1-R132C*	yes	no	Cultured under acidic conditions → no 2-HG-lactone was detected	
**All cell lines** (Fig 7 and S3)	wt or mutIDH1/2	-	-	After octly-D-2-HG treatment: Detection of 2-HG and its lactone	

Supplemental Results

Incubation of (D-)2-HG in several specimens such as serum from healthy donors and AML patients with IDH1/2 mutation, microsomes (human microsomes & S9-fraction; Life Technologies), and primary hepatocytes (100 µM and 1 mM D-2-HG, 24 h) at 37°C did not result in 2-HG-lactone production.

Recombinant IDH1/2 protein, when incubated with α-KG as substrate, showed production of 2-HG, but not 2-HG-lactone (see Figure S1). Recombinant IDH1/2 enzymes were purchased from Biomol (Hamburg, Germany; *IDH1-R132H* and WT-IDH) and Amsbio (Abingdon, UK; *IDH2-R172K*). Enzymes were incubated in a buffer adapted from Pusch et al. (2014) [1] at 37°C with gentle shaking for 30 min at a starting concentration of 1 mM α-ketoglutarate. Aliquots taken at several time points were precipitated in methanol and extracted for 2-HG/-lactone quantification by HPLC-MS/MS analysis (see method section).

Table 3. Experimental details about in vitro lactone formation.

**Specimen**	**Buffer**	**Incubation time**	**D-2-HG concentration**

**Microsomes/ S9-faction (0.5mg/ml)**	92.5 mM KH_2PO_4<br>1 mM NADPH	max 120 min	1 mM
**Serum (healthy donor)**	-	max 5 hours	100 µM/ 1 mM
**Plasma**	-	over night	100 µM/ 1 mM
**Serum (AML patients)**	-	max 5 hours	1 mM
**Recombinant enzymes**	50 mM Tris-HCl (pH 7.4)<br>0.05% BSA<br>20 µM $MgCl_2$<br>100 µM NaCl<br>1 mM αKG<br>1 mM NADPH	max 30 min	-
NADPH was always prepared freshly.			

Similarly, treatment of primary hepatocytes with 100 µM/1 mM D-2-HG or cell lines (e.g. MCF7, C7H2, see Fig. S5) with 1/5 mM D- or L-2-HG for up to 72 h did not lead to detection of significant amounts of 2-HG-lactone in either cell extract or cell culture supernatant.

We tried to establish a cell-based assay to study 2-HG-lactone formation and/or degradation. However, it was not possible to detect either activity in HCT116 cell lysates under various conditions tested. Cells were lysed by sonication in a buffer consisting of 20 mM HEPES, 25 mM KCl, 0.05/2 mM ZnCl2 and further optional components such as reducing agents (GSH, NADPH, DTT), N-dodecyl β-D-maltoside (0.5%) and 2 mM CaCl2. Also, different pH-values of the reaction mixture were tested. Either the conditions tested did not favor lactone formation or a cofactor was missing. It is also possible that lactone formation is dependent on, e.g. intact (intra)cellular organelles, which we destroyed by cell lysis. Membrane transport can also not be imitated in cell homogenates. However, the assumption that 2-HG-lactone is the transport form of 2-HG would fit the finding of higher extra- than intracellular 2-HG-lactone levels.

Supplemental Figures

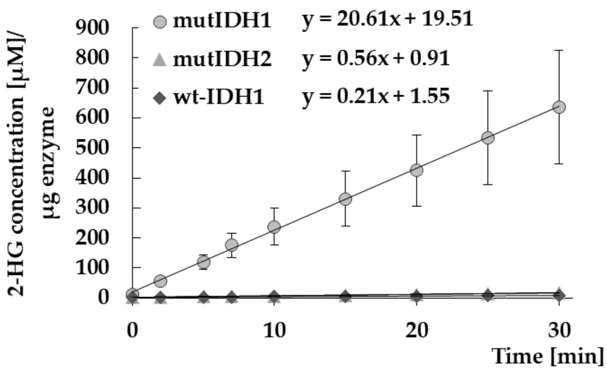

Figure 1. Enzyme assay using recombinant WT-IDH1 and mutIDH1/2 and α-ketoglutarate as substrate. 2-HG concentrations increased in a linear fashion with higher production rates for the mutated IDH1. However, 2-HG-lactone could not be detected in any of the experiments.

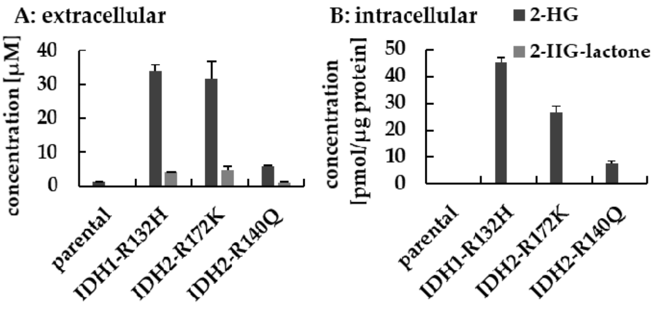

Figure 2. Extracellular and intracellular 2-HG and 2-HG-lactone concentrations in the HCT116 panel determined by HPLC-MS/MS.

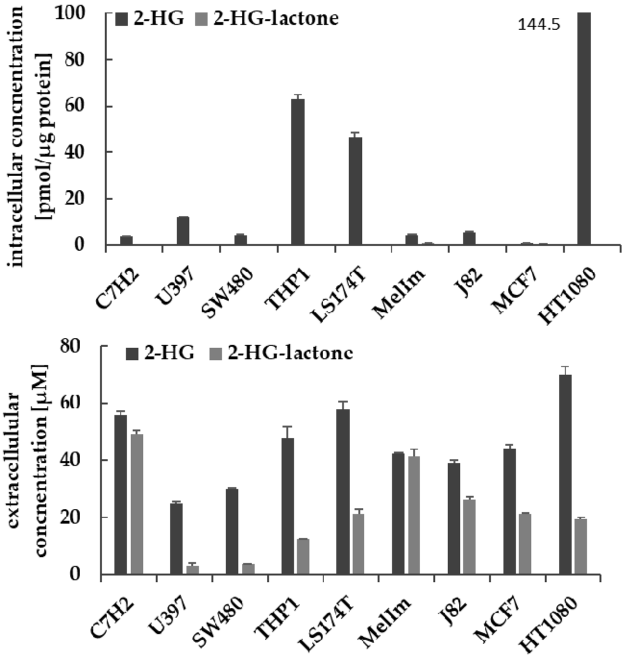

Figure 3. 2-HG and 2-HG–lactone levels in supernatants and cell extracts of octyl-2-HG treated cells. Cells were treated with 100 µM octyl-2-HG for 24 h (n=3). (Cell lines are of the following origin: C7H2 - acute lymphoblastic T leukemia, U397 and THP1 - monocytic leukemia, SW480 and LS174T - colorectal adenocarcinoma, MelIm - melanoma, J82 - bladder cancer, MCF7 - breast cancer, HT1080 (*IDH1-R132C*) - fibrosarcoma). All but HT1080 express *WT-IDH1/2*.

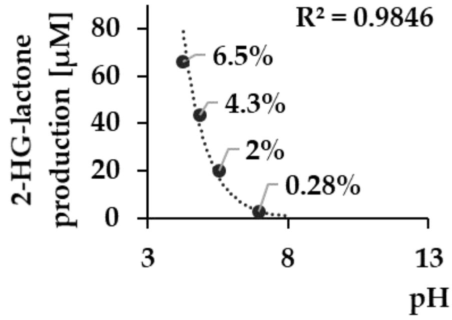

Figure 4. Spontaneous, non-enzymatic 2-HG lactonization under acidified conditions. 1 mM D-2-HG was incubated in HEPES-buffer (20 mM) acidified with HCl to different pH values (at 37°C, 29 h, n=2 per data point) and formed 2-HG-lactone was quantified.

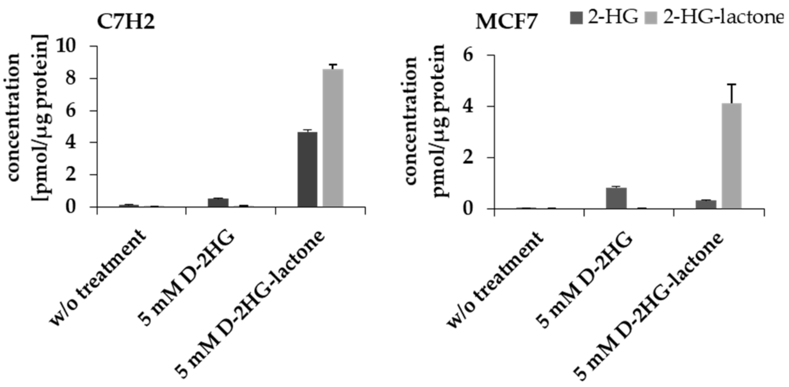

Figure 5. Intracellular concentrations of 2-HG and its lactone in C7H2 (acute lymphoblastic T leukemia) and MCF7 (breast cancer) cells (both wt-IDH1/2) after treatment with D-2-HG or D-2-HG-lactone, respectively, for 72 h. Both metabolites are detected intracellularly as they are imported. However, there is no significant lactonization in case of D-2-HG treatment. Furthermore, uptake of the two metabolites and intracellular hydrolysis of 2-HG-lactone (yielding 2-HG) inside the cell seem to be cell line specific.

Reference

1. Pusch, S.; Schweizer, L.; Beck, A.-C.; Lehmler, J.-M.; Weissert, S.; Balss, J.; Miller, A.K.; von Deimling, A. D-2-Hydroxyglutarate producing neo-enzymatic activity inversely correlates with frequency of the type of isocitrate dehydrogenase 1 mutations found in glioma. Acta Neuropath. Commun. 2014, 2, 19. doi: 10.1186/2051-5960-2-19.
